# Editorial: Alterations in brain structure and cognitive function caused by cerebrovascular diseases, volume II

**DOI:** 10.3389/fnhum.2026.1909334

**Published:** 2026-07-22

**Authors:** Xun Ye, Yuanli Zhao, Shihao He

**Affiliations:** 1 Department of Neurosurgery, Beijing Tiantan Hospital, Capital Medical University, Beijing, China; 2 China National Clinical Research Center for Neurological Diseases, Beijing, China; 3 Department of Neurosurgery, Peking Union Medical College Hospital, Peking Union Medical College and Chinese Academy of Medical Sciences, Beijing, China; 4 Department of Pathology, Johns Hopkins School of Medicine, Baltimore, MD, United States

**Keywords:** brain structure, cerebrovascular diseases, cognitive function, intracranial hemorrhage, stroke, vascular malformations

This Research Topic examines how cerebrovascular diseases are related to brain structure, cognitive performance, and functional recovery. The nine articles cover ischemic stroke, intracranial hemorrhage, cerebral venous disease, cerebral small vessel disease, vascular malformations, developmental vascular anomalies, and arterial dissection. Across these different conditions, a shared question is why similar vascular diagnoses can lead to different cognitive and functional trajectories. The articles approach this question through perfusion or venous drainage, structural imaging, systemic biological status, mood symptoms, clinical phenotype, and practical markers for follow-up and management.

## Post-stroke cognition in daily context

Zhang et al. examined elderly patients with ischemic stroke using the Montreal Cognitive Assessment, Life-Space Assessment, and Hospital Anxiety and Depression Scale. Cognitive function was positively associated with living space and negatively associated with anxiety and depression. Anxiety and depression also partly mediated the relationship between restricted living space and poorer cognition, both independently and in sequence. The study is cross-sectional, so its findings should be read as associations rather than intervention evidence. Its contribution is more specific: it places post-stroke cognition within the patient's daily activity range and emotional state, rather than treating cognition as a direct consequence of the infarct alone.

Liao et al. focused on serum uric acid to serum creatinine ratio (SUA/SCr) in acute ischemic stroke and early-onset post-stroke cognitive impairment (PSCI). Lower SUA/SCr was independently associated with PSCI assessed within 2 weeks after stroke. The result supports further testing of routine metabolic indices as early cognitive risk markers, but its predictive strength and clinical thresholds require validation before clinical use.

## Laboratory, imaging, and perioperative risk markers

Luo et al. extended this biomarker-oriented discussion to cerebral small vessel disease (CSVD). In a cross-sectional cohort, blood urea nitrogen to albumin ratio (BAR) was independently associated with CSVD and with ischemic imaging markers, including white matter hyperintensities and enlarged perivascular spaces, but not lacunes. This finding connects an accessible laboratory index with MRI-defined structural injury. The absence of an association with lacunes narrows the interpretation of BAR, suggesting that its relationship with CSVD may differ across imaging features. BAR is best presented as a laboratory marker associated with selected CSVD imaging findings, with its clinical use still requiring prospective validation.

Yang et al. investigated surgically treated patients with spontaneous intracranial hemorrhage stratified by type 2 diabetes mellitus (DM). In 688 patients, DM was associated with higher rates of post-operative major adverse cerebrovascular events, including ischemic and hemorrhagic events, as well as 30-day mortality after adjustment for confounders. Li et al. focused on basal ganglia hemorrhage and perioperative hemoglobin (HB) indices. Lower preoperative, post-operative, and mean HB levels were independently associated with unfavorable 6-month functional outcomes, and an HB-integrated model showed stronger predictive performance than single HB indicators. Read together, these studies support perioperative assessment that considers vascular comorbidity, dynamic laboratory measures, and functional endpoints, rather than hematoma location or volume alone (Li et al.; Yang et al.).

## Venous and structural phenotypes

Lai and Hu examined cerebral venous sinus thrombosis through acute-phase clinical phenotyping. They classified patients into benign intracranial hypertension, focal brain injury, and diffuse brain injury phenotypes, and reported a stepwise worsening in cognitive impairment, depressive symptoms, and return to work. The diffuse brain injury phenotype was particularly associated with poor long-term outcomes, suggesting that early bedside phenotype may help identify patients who require closer cognitive and functional follow-up.

Xiang et al. provided a case-based perspective by reporting superior sagittal sinus occlusion combined with dural arteriovenous fistula, presenting with involuntary movements, seizures, cognitive decline, and Fahr-like intracranial calcification. Digital subtraction angiography confirmed the diagnosis. This case cannot establish a general mechanism, but it illustrates how venous hypertension, abnormal drainage, seizure activity, and structural imaging findings may converge in unusual cognitive and behavioral presentations.

## Atypical vascular presentations and multimodal diagnosis

Huang et al. reported a rare case of congenital Dyke-Davidoff-Masson syndrome (DDMS) associated with hypoplastic left posterior cerebral artery (PCA) and ipsilateral fetal-type posterior communicating artery. The patient had absence seizures, preserved motor function, and occipitotemporal cognitive deficits, while multimodal imaging showed cerebral hemiatrophy, abnormal posterior circulation anatomy, and chronic PCA-territory hypoperfusion. As a single case, it should be framed as a possible posterior-circulation variant of DDMS rather than definitive evidence for a new disease subtype.

Butnariu et al. described internal carotid artery dissection presenting with hemilingual edema, occipital headache, and pulsatile tinnitus. MRI and computed tomography angiography showed a subintimal hematoma, severe luminal stenosis, and a small subacute ischemic lesion. Alongside the DDMS case, this report is mainly relevant to diagnosis: it supports multimodal vascular imaging when atypical neurological symptoms are accompanied by unexplained structural, perfusion, or arterial-wall abnormalities.

## Conclusion

The Research Topic points to a limitation of lesion-centered assessment: vascular anatomy and lesion burden alone do not fully account for cognitive and functional heterogeneity after cerebrovascular disease. The next step is not simply to validate single markers, but to evaluate cognitive testing, MRI or perfusion markers, laboratory indices, clinical phenotypes, and functional endpoints in shared prospective datasets. This would make it easier to separate markers useful for surveillance from factors that can be targeted through rehabilitation planning, perioperative management, or long-term cognitive care.

